# Diagnostic Work-Up of Neurological Syndromes in a Rural African Setting: Knowledge, Attitudes and Practices of Health Care Providers

**DOI:** 10.1371/journal.pone.0110167

**Published:** 2014-10-23

**Authors:** Alain Mpanya, Marleen Boelaert, Sylvain Baloji, Junior Matangila, Symphorien Lubanza, Emmanuel Bottieau, François Chappuis, Pascal Lutumba, David Hendrickx

**Affiliations:** 1 Division de la Recherche, Programme National de Lutte contre la Trypanosomiase Humaine Africain, Kinshasa, DR Congo; 2 Department of Public Health, Institute of Tropical Medicine, Antwerp, Belgium; 3 Sciences de la Santé, Université Pédagogique Nationale, Kinshasa, DR Congo; 4 Département de Médecine Tropical, Université de Kinshasa, Kinshasa, DR Congo; 5 Département d'Anthropologie, Université de Kinshasa, Kinshasa, DR Congo; 6 Department of Clinical Sciences, Institute of Tropical Medicine, Antwerp, Belgium; 7 Division of Tropical and Humanitarian Medicine, University Hospitals of Geneva, Geneva, Switzerland; 8 Departement d'Epidémiologie, Institut Nationale de Recherche Biomédicale, Kinshasa, DR Congo; 9 Telethon Kids Institute, University of Western Australia, Perth, Western Australia, Australia; Washington University, United States of America

## Abstract

**Background:**

Neurological disorders of infectious origin are common in rural sub-Saharan Africa and usually have serious consequences. Unfortunately, these syndromes are often poorly documented for lack of diagnostic tools. Clinical management of these diseases is a major challenge in under-equipped rural health centers and hospitals. We documented health care provider knowledge, attitudes and practices related to this syndrome in two rural health zones in Bandundu Province, Democratic Republic of Congo.

**Methods:**

We used a qualitative research approach combining observation, in-depth interviews and focus group discussions. We observed 20 patient-provider contacts related to a neurological syndrome, conducted 12 individual interviews and 4 focus group discussions with care providers. All interviews were audiotaped and the transcripts were analyzed with the software ATLAS.ti.

**Results:**

Care providers in this region usually limit their diagnostic work-up to clinical examination primarily because of the financial hurdles in this entirely out-of-pocket payment system. The patients prefer to purchase drugs rather than diagnostic tests. Moreover the general lack of diagnostic tools and the representation of the clinician as a “diviner” do not enhance any use of laboratory or other diagnostic methods.

**Conclusion:**

Innovation in diagnostic technology for neurological disorders is badly needed in Central-Africa, but its uptake in clinical practice will only be a success if tools are simple, affordable and embedded in a patient-centered approach.

## Introduction

The frequency of neurological disorders and their etiology is poorly documented in low-resource settings, particularly in sub-Saharan Africa. The scarce data suggest however that the burden is substantial, since neurological disorders accounted for 7–24% of all admissions in African hospitals in studies conducted in the past 20 years [1]–[4]. In these few studies, infections of the central nervous system (CNS) were suspected in about one third of patients admitted with neurological disorders, although no specific etiology was found in nearly half of the cases [2]. In Central Africa for example, neurological disorders may be due to infections such as Human African trypanosomiasis (HAT), cerebral malaria, bacterial meningitis, tuberculous meningitis, neurosyphilis, cryptococcal meningitis or encephalitis due to *Toxoplasma* [5]. If such conditions are not timely treated, death or serious sequelae occur frequently [6], while early specific treatment may substantially improve the outcome. In resource-limited settings such severe and treatable conditions should be targeted as a priority in the process of clinical decision making in order to prevent complications and death [7]. Unfortunately, most neuro-infections present with non-specific symptoms in their early stages, leading to significant delays in diagnosis [8], [9]. The management of neurological disorders is a major challenge in the under-equipped rural health centers and hospitals of the Democratic Republic of Congo (DRC), since the potential infectious etiologies are extremely diverse and diagnostic facilities (imaging, laboratory) are dramatically lacking. There is a need for simple, affordable, robust and reliable diagnostic tools to improve the quality of care for these patients. As part of a wider research program that focuses on innovative diagnostic technologies to integrate in a syndromic approach to neurological conditions, we set out to study the perspective of the local clinicians working in low-resource settings. We surmise that a better understanding of the complex reality in these rural areas may optimize the uptake and effectiveness of the new diagnostic technologies and clinical algorithms that are being developed. The study aims to describe the knowledge, attitudes and current practices of clinicians when faced with a patient presenting with a neurological condition in a rural area of DRC where access to diagnostic tools is limited.

## Methodology

### Definition of the neurological syndrome

The following symptoms were used to define what constitutes a neurological syndrome in the context of this study: new-onset seizure, altered state of consciousness, sensory-motor deficits (central or peripheral), walking disturbances not due to orthopaedic causes, and/or headache (with meningeal sign, or daily, crescendo, for which a lumbar puncture is clinically justified).

### Research area and study population

**Figure 1:**
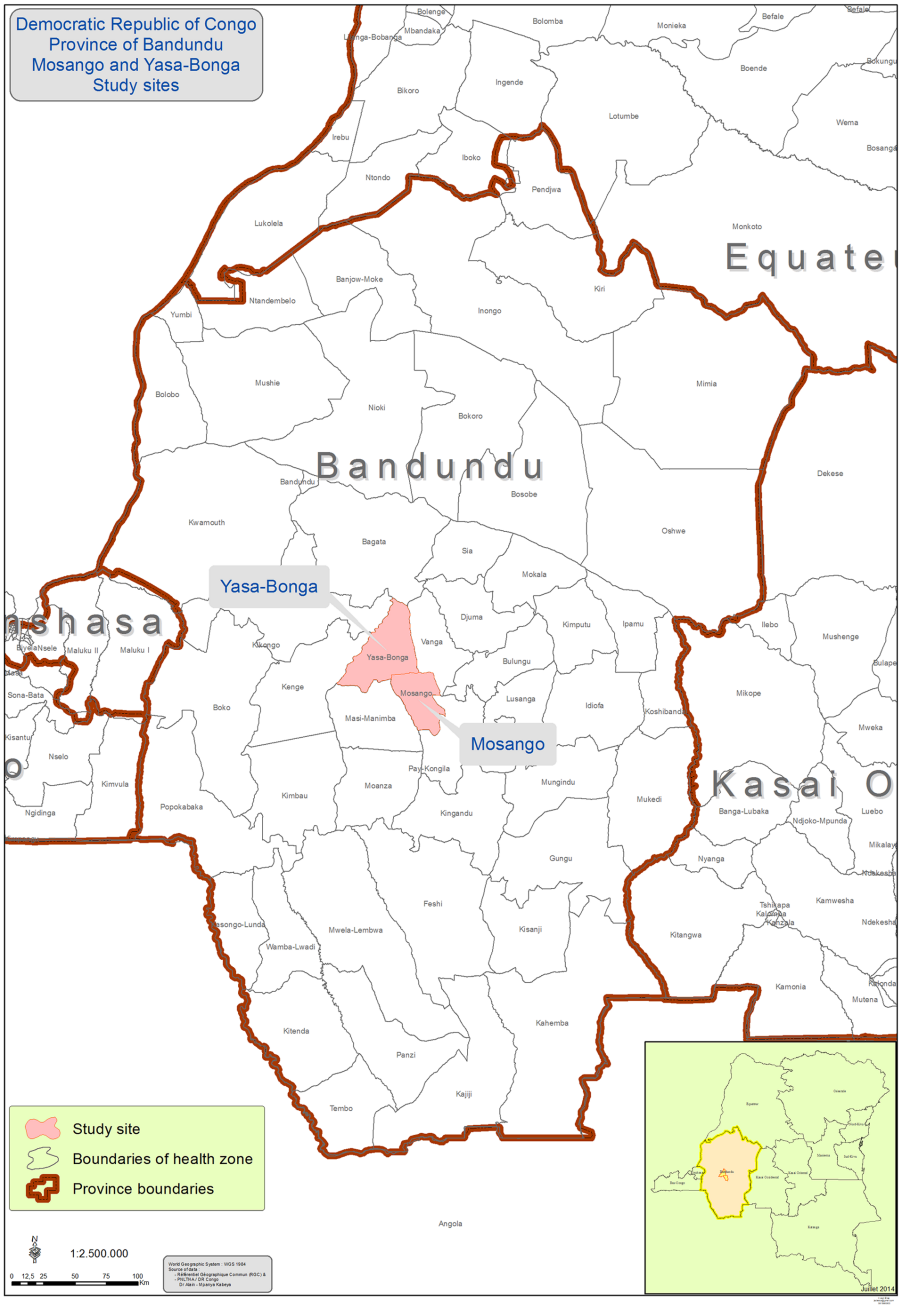
Location of the study sites.

This study was conducted in two rural health areas (Mosango and YasaBonga) in Bandundu province in the DRC (see Figure 1). Bandundu Province is one of the eleven provinces of DRC, located in the west near the Congo-Kinshasa and Bas-Congo provinces. Its total area is about 300,000 km^2^ and the population stands at over 5,000,000 inhabitants. Outside of the two main cities, Kikwit and Bandundu, the province is completely rural and transport is difficult, while a large proportion of the population remains poverty-stricken. Furthermore, health services are extremely fragile, under-equipped and utilization is very low.

The study population consisted of health care providers working at one of the study area's primary healthcare centers or one of its two general referral hospitals (Mosango and YasaBonga). The following two groups of health care providers were therefore the focus of the study: i) physicians performing out-patient consultations in the hospitals of Mosango and YasaBonga, and ii) the nurses in charge of daily consultations in the health centers of Mosango and YasaBonga.

### Study design and implementation

**Figure 2:**
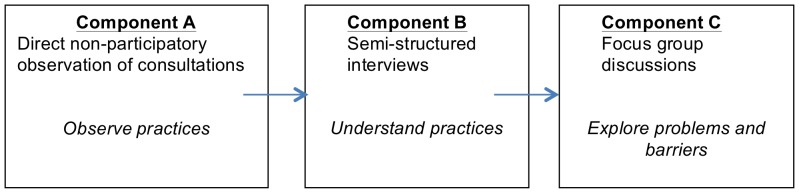
Sequence of research instruments

This study adopts a qualitative approach, based on the theoretical frameworks of phenomenology and grounded theory [10], [11]. These employ an inductive research strategy that is intended to gain an informed understanding of certain phenomena from the perspective of those experiencing them. We combined non-participant observations, individual interviews and focus group discussions (FGDs) to document the current practices of health care providers working in rural areas when faced with a neurological disorder. This approach allowed for triangulation, while the sequential nature of the study design also introduced a degree of reflexivity that allowed us to adapt the interview and focus group discussion question guides in line with the outcomes of prior study components. Although some healthcare providers participated in all three study phases, this was not a prerequisite for our study. Data collection lasted two months (November and December 2011), starting with the non-participatory observations, followed by interviews and FGDs (see Figure 2). The total number of observations, interviews and focus group discussions is summarised in Table 1.

**Table 1:** Number of observations, interviews and focus groups by category of respondents.

N°	Category	Observations	Interviews	Focus groups
1	Hospital clinicians	10	6	2
2	Health centre nurses	10	6	2
	**Total**	**20**	**12**	**4**

### Direct non-participant observation

We observed a total of 20 consultations related to the neurological syndrome of which 10 were conducted by physicians of the general referral hospital and 10 by health center nurses (for details, see Table S1). The observations were conducted in three purposefully selected health centers and a general referral hospital in each of the two health zones (Mosango and YasaBonga). These observations served to document current practices and inform the development of our interview and focus group discussion question guides. Consultations by hospital clinicians were observed by a researcher with a degree in medicine, while consultations performed by nurses at the health center were observed by a researcher with a background in nursing.

The same standardized observation check list was used throughout all observed consultations (see Text S1). The checklist included comment sections that the observing researchers used to summarise the consultation and elaborate on observations that were of particular interest. The resulting qualitative data was included in our analysis. In order to minimise observer effect bias, the observations were planned over one month, during which the observing researcher also observed (but did not document) non-neurological consultations.

### Semi-structured interviews

The interviews were conducted approximately three weeks after the observations of consultations. This allowed investigators to adapt the question guide based on the outcomes of those observations. Each interview started with a clinical vignette -a hypothetical case of a patient presenting with the neurological syndrome- to better understand the logic behind the healthcare provider's consultation process (see Text S2). This was followed by a semi-structured interview to investigate care practices in the management of neurological syndrome cases (for the question guide, see Text S3). We purposively selected and interviewed three clinicians and three nurses in each health zone, a total of 12 interviews (for details, see Table S2). The interviews were pre-tested, conducted in French and recorded on a digital medium.

### Focus group discussions

We conducted four focus group discussions: two with hospital physicians and two with health center nurses (for details, see Table S3). Each group consisted of seven purposively selected participants. Since only four clinicians worked at the Mosango hospital, we recruited an additional three participants from the neighbouring Masimaniba health zone hospital for that particular FGD in order to ensure a dynamic discussion. As the Masimaniba and Mosango health zones are very similar in terms of their geographic, socio-economic and epidemiological settings, we did not expect this decision to introduce any bias into our study. The discussions were geared particularly towards exploring the challenges that healthcare providers perceived in association with the management of neurological syndrome cases (for the question guide, see Text S4). The focus group discussions lasted an hour on average, were conducted in French on the basis of a pre-tested question guide (informed by the outcomes of the observation and interview study components), and were recorded on a digital medium.

### Data analysis

Interviews and FGDs were recorded on a digital medium, transcribed verbatim and converted into MS Word documents. The transcripts were then imported into ATLAS.ti, a computer application that supports the systematic analysis of qualitative data.

The central concept of qualitative data analysis is referred to as ‘coding’, a systematic and iterative process where the researcher reduces all the available transcript data into meaningful segments of text (or codes) [12]. These text segments are then labeled and organised into a coding tree, which provides a visual representation of the data's structure. Our overall coding strategy for this study was developed on the basis of the interview and FGD question guides. We developed an initial coding tree in line with the topics that were introduced in each of those questions. We then applied an iterative bottom-up approach while reading through each of the transcripts several times and added new codes and code branches as new items emerged from the data. The resulting coding tree was then discussed and finalised by several members of the research team with an expertise in qualitative research methods. The general structure of our coding process is provided in annex (see Text S5).

### Ethical statement

The study protocol was approved by the ethics committee of the School of Public Health of the University of Kinshasa in DR Congo and by the Institute of Tropical Medicine, Antwerp, Belgium. We obtained the authorization from the Kwilu health district (which includes Mosango and YasaBonga health zones) to perform the study in each of the two health zones. In order to avoid bias, the informed consent for the observational study component of the clinicians' consultations consisted of a two-tiered process: an initial oral consent before starting the observations and a written consent after finalising the observations in which the study's specific focus on the neurological syndrome was specified. This two-stepped process was considered necessary in terms of minimising observer bias as much as possible. For the individual interviews and focus group discussions, oral consent was obtained before starting to record the discussions. Participants were informed about the voluntary nature of their participation. Anonymity of the participants was guaranteed and no personal details were recorded.

## Results

### Knowledge of the neurological syndrome

The care providers at the health centers and hospitals in these rural areas are quite familiar with the neurological syndrome as a clinical presentation. They understand the syndrome as a set of signs and symptoms that reflect an impairment of the nervous system. The most often evoked signs are seizures, coma, decreased consciousness, agitation, logorrhea, walking disturbances, severe headache and meningeal signs such as neck stiffness. "*Coma, convulsions, motor or sensory deficits,…, this list is not exhaustive, there are several clinical expressions that point to the concept of a neurological syndrome*." (FGD1). Physicians as well as nurses say that several infectious diseases can cause the neurological syndrome in their community. The most often cited causes are malaria, HAT, tuberculosis, HIV and bacterial meningitis. "*Among the infectious diseases that cause neurological problems in our setting we can quote malaria, trypanosomiasis, meningitis, leprosy*." (FGD4). During our observations of neurology-related consultations health care providers would include the same list of diseases in their differential diagnoses "*Diagnostic hypotheses are first of all trypanosomiasis, secondly meningitis and thirdly cerebral malaria.*"(OBS8). Care providers also say that the neurological syndrome may equally be due to various forms of intoxication, such as alcohol, which must therefore always be ruled out. "*There are products that are toxic, that are irritable, which may generate irritability in the patient, even behavioral problems*" (FGD2). Care providers are conscious of the possibility that the neurological syndrome may be the result of a newly emerging infectious disease that may not yet have been documented in the area. They acknowledge that such diseases may go unnoticed in their setting, since they usually limit their differential diagnosis to well-known pathogens and given that laboratory facilities are very poor. Overall, there seems to be no ascertainable difference in general knowledge concerning the neurological syndrome between the health center nurses and hospital physicians in the rural health zones in which our study took place. Differences exist rather in the respective approaches they take to the diagnosis and clinical management of neurological syndrome cases.

### Diagnosis and clinical management

The diagnosis of a neurological syndrome case is usually made at the level of the health zone's referral hospital. At the health center level, “*ordinogrammes*” are provided by the Ministry of Health. These are manuals filled with flow charts that dictate the course of action to be taken for a specific syndrome. In the case of a patient presenting with symptoms associated with the neurological syndrome, the nurse is instructed to promptly refer the patient to the health zone's referral hospital. This decision is solely based on clinical signs, as the relevant flow chart does not call for any confirmatory examinations to be done by the nurse. "*Our diagnosis is clinical only. When there is already a behavioral disorder, convulsion, temporal-spatial disorientation, we must refer the patient.*" (INT6). Health centre nurses put forward a presumptive diagnosis and prescribe symptomatic treatment before referring the patient to the hospital for confirmation and case management. "*The health center does not have a laboratory. The nurse does not prescribe tests and refers the case after giving diazepam.*"(OBS20) In the case of a suspicion of sleeping sickness, the nurse must link the patient up with the specialized sleeping sickness program that operates with mobile teams and visits each village once a year. "*The nurse requests the CATT test that the mobile teams will come to make, he must make a note of the identity of the patient and wait until the visit of the mobile team.*"(OBS17). However, there are a small number of health centers (and hospitals) that can offer more diagnostic services-usually under the form of rapid diagnostic tests- thanks to the support of specialized vertical programs. These may provide diagnostic tools for various infectious diseases such as malaria, HIV/AIDS and also in some cases HAT. "*No microscope and no laboratory examinations. Only Rapid Diagnostic Tests for malaria.*" (OBS19).

For hospital clinicians, the signs suggestive of the neurological syndrome are considered too clear to miss. A patient presenting with the neurological syndrome accompanied by a fever leads them to believe the cause is of infectious origin. "*Regarding the process of diagnosis, sometimes it is easy, in the case the complaint begins with a fever. If that is not the case, then we say the field is wide open.*" (FGD1). In the absence of fever, clinicians indicate a need for additional tests, which they feel patients might be unable to cope with financially. Because of this, in practice the presumptive diagnosis at the referral hospital level remains largely informed by clinical signs, symptoms and the epidemiological context of the area. "*Even if there is no fever, you can orient yourself towards a certain pathology based on what we know about the onset of the disease and the area in which the patient lives.*" (FGD1). Hospital clinicians rarely obtain a definitive diagnosis before establishing a medical treatment, for which they usually work on the basis of a presumptive diagnosis. Even if the clinician requests laboratory tests to be done, the patient will be put on a treatment regime immediately after the sample collection, without waiting for the laboratory results "… *when you just wait for the definitive diagnosis from the laboratory, it makes it really difficult, so at times we prescribe a treatment as such.*" (FGD1). Healthcare providers indicate how delaying treatment pending test results -even if it is just for one day- is considered to be poor clinical practice by the patient and the wider community. Healthcare providers are convinced that the community considers it to be inconceivable that a patient spends any amount of time in the hospital without being put on any kind of treatment regime. That is why hospital clinicians tend to prescribe a treatment immediately instead of awaiting the laboratory test outcomes. "*Keeping the patient in the hospital without providing treatment for 24 hours, I assure you that the hospital would quickly empty. They will say ‘I have been here since morning, no pills, nothing'. The next time the patient will simply not come back.*” (INT15).

The most commonly performed laboratory tests requested by hospital clinicians are thick film (GE), hemoglobin (Hb), examination of stool and urine, and what is known in French as “un bilan inflammatoire”, a panel of tests composed of the erythrocyte sedimentation rate (ESR) and a total and differential white blood cell count (WBC). These examinations are requested almost routinely in every patient that presents to the hospital. "*For example, the ‘bilan inflammatoire’, I don't think there is any laboratory request form here which does not include a request to have a ‘bilan inflammatoire’ performed*."(INT10). Clinicians say that these routine tests are easily feasible in their setting and are also considered to be affordable by the community. "*We request them because they are within our reach, we can do them here. And their cost is affordable for all patients.*"(INT9).

### Perceived barriers to diagnosis

Having laboratory tests done, especially those required for an etiological diagnosis, is considered problematic in many cases, even at the referral hospital level. The most often reported barriers to diagnosis are related to the limited financial capabilities of the community, a lack of diagnostic tools and laboratory reagents, the long turn-around-time of laboratory test results and the community's perceptions and expectations associated with the care provider.

Firstly, the necessary laboratory tests required to adequately resolve a differential diagnosis and identify the etiology might simply not be available to the hospital clinicians. A number of essential tests, such as bacteriological culture, are lacking. "…*to identify the germ is difficult because there you may have to go to culture or take a blood culture in a special medium and that is where the real problem begins.*" (FGD1). The observations performed in the hospitals confirm this: "*Results obtained beyond the clinical assessment are very limited, some tests cannot be done for lack of reagents.*" (OBS5). If meningitis is suspected, then a lumbar puncture is requested to assess the appearance of the cerebrospinal fluid (CSF), and both cytology and Gram staining would also be requested. However, biochemistry of the CSF is rarely performed and culture is impossible in this setting. As a result hospital clinicians may miss less common but equally serious pathologies such as tuberculous meningitis. "*We're not sure, really, we do not pose any diagnostic certainty for lack of diagnostic tools.*"(INT13). At times the skills of the laboratory technicians are the limiting factor and generally the lack of electricity is also a major constraint for the realization of many laboratory tests.

Secondly, the capacity to have any laboratory test performed at the referral hospital level depends not only on the availability of the relevant laboratory test, but also on the purchasing power of the patient. Therefore the low standard of living of these rural communities is considered a serious impediment for laboratory investigations according to the healthcare providers. This issue is exacerbated by the choice hospital clinicians regularly need to make between having additional tests done on the one hand, and -on the other- ensuring the patient remains able to pay for any drug prescription or treatment regime that might follow."… *despite the good will to ask for investigations (laboratory tests), we wonder, will they be within reach of the purse of the patient, will the lab conduct them promptly*?"(FGD3). "*We request them (laboratory tests) based on the clinical presentation, but also depending on the (financial) resources of the patient* " (INT 9). Healthcare providers say it is useless to request a laboratory test to be done that the patient will not be able to pay for. Financial considerations also have further repercussions, since the hospital laboratory would only order reagents that patients will be able to pay for. "*Indeed, the institution purchases reagents based on the context here. We cannot bring in reagents for which patients will not be able to bear the cost.* "(INT15). Laboratory investigations are therefore usually kept to a strict minimum, although the panel of requested laboratory tests may be expanded in the case of patients that are financially better-off *«In the case of patients who have more money, I can add other investigations*" (INT9).

A third barrier associated with the diagnosis of neurological syndrome cases relates to the long delay that may occur between requesting a laboratory test and receiving the results. In emergency cases, the laboratory may simply not be able to provide instant test results, and the clinician is often forced to proceed without them. *"We sometimes need to act quicker than we should, as we should await the results within 3 days. But in the meantime, the person arrived in very poor shape."*(FGD3). Another factor healthcare providers referred to that might cause a significant delay in having laboratory tests done was again related to financial considerations, since in some cases the delay occurs due to family members of the patient having to return to their village to find the necessary money to pay for the tests.

A final factor to consider in regard to the use of laboratory tests relates to the perceptions and expectations the community has of healthcare providers in general and clinicians in particular. Clinicians are perceived to be imbued with special knowledge and skills that instill upon them a semi-godlike status. Healthcare providers talk of patients expecting them to be able to directly identify the cause of their illness without the need for laboratory tests, much in the same way as the diviners, a group of traditional healers, would identify the causation of a disease. This explains why the community perception attaches more importance to medical prescriptions and actual treatment than to laboratory investigations. "*Patients don't consider laboratory examinations to be their business. The doctor should treat on the basis of on their complaints. It is all related to the culture.*" (INT15).

### Sources of medical information

In general, healthcare providers know of various relevant clinical reference guides and confirm their usefulness in directing the management of patients presenting with the neurological syndrome. The format and content of these guides varies depending on whether they are intended to be used at the level of the health center or the health zone's general referral hospital. As mentioned earlier, guidelines provided to the health centers in the form of 'ordinogrammes' simply recommend the nurse to refer a patient presenting with signs and symptoms associated with the neurological syndrome to the health zone's hospital. At the hospital level, clinicians are provided with clinical management protocols specific to certain infectious diseases that are common in the area. The documents may vary from just a few pages up to a hundred. "*We have separate protocols for tuberculosis, malaria, trypanosomiasis and for HIV.* "(INT16). On the other hand, referral hospital clinicians indicate that there is currently no clinical guideline or other kind of resource that provides comprehensive support for the management of patients presenting with the neurological syndrome. "*To be honest, no, I am not aware of such a reference document, for each pathology separately maybe yes, but for the neurological syndrome as a whole, no.* "(INT15).

Healthcare providers perceive the kind of reference documents listed above to be useful and necessary tools for obtaining information that they require to improve their clinical management practices in general, but do not see them as particularly useful tools to use during patient consultations with the aim of guiding their clinical diagnosis. Health care providers, both at the hospital and health center level, cite several reasons for this. First of all, healthcare providers are concerned that using any kind of reference document in the presence of the patient might undermine the trust patients have in the capabilities of the nurse or doctor that they are consulting with. Healthcare providers are wary of being perceived as ignorant and incompetent. This is especially the case for clinicians, given the semi-deity-like status that the community bestows upon them. "*When you consult the ordinogrammes (clinical flow charts) in the presence of patients, you disturb their psychology. They might say you do not know how to take care of them and you automatically lose their trust*"(FGD4). "*The patients who come to see the doctor or nurse, they see them as people who are very knowledgeable. To see them read a document in their presence, the patient would lose their confidence in you. It is the culture here*." (INT8). "*In the culture, the mentality here in this area, the doctor is perceived as a little god*." (FGD1). There is also the notion that consulting such resources in the presence of the patient amounts to cheating, a notion shared by both patients and healthcare providers themselves. "*Checking notes, I think it's a bit like cheating. No, you cannot cheat in front of the patient. If we do it, we do it when the patient is not around*."(FGD3). Logistical issues also come into play in explaining why reference documents are generally not used during patient consultations. This can be due to the lack of availability of certain resources in the consultation room. "*We do not have any protocols available in our offices. Sometimes the program sends us too few of them*."(FGD3), but healthcare providers also say they simply have no time to consult clinical management reference documents given how busy the area's health centres and hospitals generally are. There is always the pressure of time, since there are usually long lines of patients waiting to be seen. In addition, in the case of an emergency, the patient is often accompanied by a crowd of people who all expect the patient to be helped immediately. Clinicians say it is impossible to consults any clinical reference documents in such conditions. "*I do not do it (consult clinical resource documents) in the presence of sick because I simply do not have time.*" (FGD4).

### Referral of patients

The health centre flowcharts-or 'ordinogrammes'- instruct health centre nurses to refer cases presenting with signs and symptoms associated with the neurological syndrome to the health zone's referral hospital. As such, the health centres consider the referral to be an indicator of good practice and a performance criterion demonstrating continuity of care. "*It is a performance criterion. If a health center does not refer, it is a bad health center, and a bad nurse.*"(FGD2).

Healthcare providers distinguish several barriers that they associate with the referral of patients from the health centre to the zonal hospital. The travel distance from the village where patients live to the referral hospital is usually such that patients become discouraged and may not follow-up on the referral since they lack means of transportation. "*You can refer the patient, but he might tell you 'me, I have no means of transportation'. There are also patients who tell me 'I have no money'. Me, I referred a patient recently, I gave him a referral letter, he tore it up and returned to his village.*" (FGD2).

Another factor that affects referral is cost. Healthcare providers say medical care is perceived to be more expensive at the hospital than at the health centre. Patients are therefore reluctant to go to the hospital since they fear they will need to spend more money, not just for a consultation, but also for laboratory tests, medication and possible hospitalization. "*When referring the patient to the hospital, the patient thinks first of the money. The cost of hospitalization is not within the reach of villagers.*" (FGD4). We documented a case of a patient refusing referral because of cost considerations as part of the consultations that were observed. From the observation notes: "*He (the nurse) told the family member that referral of the patient was necessary. The drug he gave the patient is only diazepam. He prepared a transfer letter for YasaBonga, but the family refused due to lack of money.*"(OBS20).

A final barrier to the referral process that was identified by healthcare providers concerns the patient's interpretation of the act of referring itself. The need for referral might be perceived as a failure on behalf of the healthcare provider -and modern medicine- to be able to resolve the health issue. In such cases the patient's carers might consider alternate, folkloristic, reasons to explain the failure. The cause is usually sought in the patient's family and might be related to an unresolved feud or other imbalance in the patient's immediate environment. This issue would have to be resolved first before the patient would travel to the referral hospital in the hope that this will avoid an additional medical failure. "*Seeing a doctor at the hospital for them is considered to be a solution. If they return from the hospital without a solution, that is to say that there is a problem in the village, witchcraft, maybe the uncle is involved, so they say that if the healthcare provider refers, it is because there is a problem in the family.* "(INT8).

## Discussion

This study shows that the diagnostic work up of a neurological syndrome case in rural hospitals in DRC is largely based on the clinical presentation, without any laboratory or imaging confirmation. This quasi absence of diagnostic confirmation is explained by several factors; the general lack of specific and adapted diagnostic tools, the unaffordability of such diagnostic tests, the long turn-around-time between sample taking and result and last but not least the perception that the community has of the care provider, who is expected to diagnose the underlying problem much in the same way as a diviner would do. The clinicians who deliver clinical care in these rural areas say they are perceived as someone with ‘divinatory’ power capable of directly identifying the disease in a patient. This role expectation has roots in the local tradition where the disease is considered not merely as a biological phenomenon but also a social and spiritual phenomenon. In this system, the traditional healer is invested with supernatural powers and communicates with spirits to identify the sorcerer who caused the disease: someone in the community who cast a spell on the patient. Importantly, the patients and relatives consider the neurological syndrome as a supernatural phenomenon belonging to the domain of witchcraft [13]–[15]. The role concept of “diviner” is one of the factors that prevent the clinicians from consulting a reference books or guidelines in front of the patient during the consultation. This attitude of the clinicians can be explained by the fear of losing authority (being someone who needs to know all what is in textbooks) and maybe also by patterns instilled during the undergraduate training where students are expected to remember everything by heart, and opening a textbook is seen as cheating. Adapting the format of clinical management guidelines or flowcharts as to make them more acceptable for use in the presence of the patient could improve the quality of care. A digital format that may be used on a computer or other digital device might be considered, a possible solution that was suggested by a clinician during our pre-tests. On the other hand, the cost and lack of a reliable energy source may limit its use in rural areas. Current guidelines available in health centres state that nurses should refer neurological syndrome cases to the zonal hospitals. This is problematic since issues of cost and distance might lead the patient not to follow up on the referral. Furthermore, the act of referral may be perceived by the community as a sign that the illness has other, spiritual, causes that need to be resolved before seeking medical attention again.

This study used a combination of several qualitative data collection methods (observations, interviews and focus group discussions) to allow for triangulation of our findings. We only performed four focus group discussions, which might be considered a relatively low number for this kind of study. However, no additional sessions were necessary since all health centre nurses and hospital clinicians working in our study area participated in either one of the four focus group discussions we organised. Since we had exhausted our sample, we were confident data saturation had been reached. An important limitation of this study is that we did not interview members of the communities. The community attitudes and practices described here are based on the healthcare providers' perceptions of the community and may therefore not veraciously reflect the community's true attitudes and practices.

Our findings align with those of other authors who pointed to the challenges of diagnostic work-up of infectious diseases in the tropics [16]. Cohen et al. [17] showed that the diagnostic confirmation of bacterial meningitis and meningococcal cryptococcosis is very difficult in situations with limited resources. An exclusive clinical work-up of a serious and life-threatening condition such as the neurological syndrome is unfortunately very common in resource-limited settings. Palmer et al. [18] evaluated a clinical algorithm for the detection of sleeping sickness, but showed this leads to detection of mainly stage-2 patients. Several other authors have pointed out how exclusively clinical approaches lead to misdiagnosis, avoidable deaths and sequelae. Moreover it leads to an increase in the number of unnecessary prescriptions and postpones the real diagnosis [5]. An aspect that contributes to the diagnostic delays is the fact that patients are forced to consult several different providers and health facilities in their search for a cure. Hasker et al. [8] revealed in their study on the health seeking behavior of sleeping sickness patients that a median of four contacts (IQR 3^rd^- 7^th^ contact) were necessary before a confirmed diagnosis was made. This protracted search for a cure not only increases the risk of complications and irreversible neurological sequelae but also increases the cost of the whole episode for the patient and his/her family.

Poverty breeds conditions for the development and spread of infectious diseases and simultaneously prevents access to quality healthcare [19], [20]. WHO [20] estimates that more than 3 billion people live on less than $2 a day. According to Wagstaff [21] poverty is characterized by insufficient use of health services, a lack of nutrition and leads to health damaging practices. Under these conditions, it is difficult to bear the cost of health care, as a decision to visit the health centre and pay for the related costs would often come at the expense of the capacity to buy other essential goods, such as food. What is more, given their limited income, patients might have to make additional choices in regards to the various costs involved (consultation, medicine, laboratory tests), as paying for all of them might not be an option. In practice, patients decide to allocate their limited funds as much as possible to the purchase of medicines, with laboratory tests not being considered a priority (Perception and reality of the communities on the system of health care provision in the DRC, manuscript not yet published).

We conclude that the lack of affordable and practical diagnostic tools in such rural African settings is a major problem, and innovation in diagnostic technology is badly needed. The lack of simple and reliable diagnostic tools in the primary care setting too often leads to delays in diagnosis and life-saving treatment. Rapid diagnostic tests offer great potential for such settings, as demonstrated in the field of HIV/AIDS and malaria. Until very recently there was no appropriate test for screening people for sleeping sickness at the level of the health centre, while parasitological confirmation tests remain problematic in this setting [22]. Fortunately, recent innovations in the diagnostic technology for HAT show there might be cause for optimism. A rapid diagnostic test for HAT is now available [23], [24], molecular tools such as LAMP (loop-mediated isothermal amplification) are being evaluated for their potential use as confirmatory tests [25] and a proof of concept has been delivered on the use of certain biomarkers for staging of HAT [26].

In conclusion, identifying (treatable) infectious etiologies of neurological disorders can be challenging in low-resource settings in rural Africa such as the region that was the focus or our study. The technical development of relevant simple point-of-care tests is essential, but ensuring their use and effective implementation in such settings requires more. Ensuring accessibility of those tests, in terms of cost and possible local socio-cultural considerations, is just as important to address.

## Supporting Information

Table S1 **Characteristics of observed consultations.**
(DOCX)

Table S2 **Characteristics of interviews.**
(DOCX)

Table S3 **Characteristics of focus group discussions.**
(DOCX)

Text S1 **Check-list used for observing consultations.**
(DOCX)

Text S2 **Clinical vignette used in interviews.**
(DOCX)

Text S3 **Interview question guide.**
(DOCX)

Text S4 **Focus group discussion question guide.**
(DOCX)

Text S5 **Coding structure.**
(DOCX)
