# Supplementary material for: Diagnostic Work-Up of Neurological Syndromes in a Rural African Setting: Knowledge, Attitudes and Practices of Health Care Providers
Source: PLoS One. 2014 Oct 23;9(10):e110167. doi: 10.1371/journal.pone.0110167 (PMC4207747; doi:10.1371/journal.pone.0110167)
Supplement: Table S1 — Characteristics of observed consultations. (DOCX) [file pone.0110167.s001.docx]

**Table S 1:** Characteristics of observed consultations

| **Observations nr** | **Health zone** | **Health facility name** | **Health facility type** | **Health provider type** |
| --- | --- | --- | --- | --- |
| OBS 1 | Mosango | Mosango | General reference hospital | Physician |
| OBS 2 | Mosango | Mosango | General reference hospital | Physician |
| OBS 3 | Mosango | Mosango | General reference hospital | Physician |
| OBS 4 | Mosango | Mosango | General reference hospital | Physician |
| OBS 5 | Mosango | Mosango | General reference hospital | Physician |
| OBS 6 | YasaBonga | YasaBonga | General reference hospital | Physician |
| OBS 7 | YasaBonga | YasaBonga | General reference hospital | Physician |
| OBS 8 | YasaBonga | YasaBonga | General reference hospital | Physician |
| OBS 9 | YasaBonga | YasaBonga | General reference hospital | Physician |
| OBS 10 | YasaBonga | YasaBonga | General reference hospital | Physician |
| OBS 11 | Mosango | Mosango | Primary health centre | Head nurse |
| OBS 12 | Mosango | Muluma | Primary health centre | Head nurse |
| OBS 13 | Mosango | Muluma | Primary health centre | Head nurse |
| OBS 14 | Mosango | Tumikia | Primary health centre | Head nurse |
| OBS 15 | Mosango | Tumikia | Primary health centre | Head nurse |
| OBS 16 | YasaBonga | Yasa | Primary health centre | Head nurse |
| OBS 17 | YasaBonga | Fula | Primary health centre | Head nurse |
| OBS 18 | YasaBonga | Fula | Primary health centre | Head nurse |
| OBS 19 | YasaBonga | Kwaya | Primary health centre | Head nurse |
| OBS 20 | YasaBonga | Kwaya | Primary health centre | Head nurse |
