# Supplementary material for: Diagnostic Work-Up of Neurological Syndromes in a Rural African Setting: Knowledge, Attitudes and Practices of Health Care Providers
Source: PLoS One. 2014 Oct 23;9(10):e110167. doi: 10.1371/journal.pone.0110167 (PMC4207747; doi:10.1371/journal.pone.0110167)
Supplement: Table S2 — Characteristics of interviews. (DOCX) [file pone.0110167.s002.docx]

**Table S 2:** Characteristics of interviews

| **interviews nr** | **Health zone** | **Health facility name** | **Health facility type** | **Health provider type** |
| --- | --- | --- | --- | --- |
| INT 5 | Mosango | Mosango | Primary health centre | Head nurse |
| INT 6 | Mosango | Kasaï | Primary health centre | Head nurse |
| INT 7 | Mosango | Muluma | Primary health centre | Head nurse |
| INT 8 | Mosango | Mosango | General reference hospital | Physician |
| INT 9 | Mosango | Mosango | General reference hospital | Physician |
| INT 10 | Mosango | Mosango | General reference hospital | Physician |
| INT 11 | YasaBonga | Yasa | Primary health centre | Head nurse |
| INT 12 | YasaBonga | Mukengi | Primary health centre | Head nurse |
| INT 13 | YasaBonga | Kwaya | Primary health centre | Head nurse |
| INT 14 | YasaBonga | YasaBonga | General reference hospital | Physician |
| INT 15 | YasaBonga | YasaBonga | General reference hospital | Physician |
| INT 16 | YasaBonga | YasaBonga | General reference hospital | Physician |
