# Supplementary material for: Diagnostic Work-Up of Neurological Syndromes in a Rural African Setting: Knowledge, Attitudes and Practices of Health Care Providers
Source: PLoS One. 2014 Oct 23;9(10):e110167. doi: 10.1371/journal.pone.0110167 (PMC4207747; doi:10.1371/journal.pone.0110167)
Supplement: Table S3 — Characteristics of focus group discussions. (DOCX) [file pone.0110167.s003.docx]

**Table S 3:** Characteristics of focus group discussions

| **FGD nr** | **Health zone** | **Health facility type** | **Health provider type** | **Number of participants** |
| --- | --- | --- | --- | --- |
| FG 1 | Mosango | General reference hospital | Physician | 7 |
| FG 2 | Mosango | Primary health centre | Head nurse | 7 |
| FG 3 | YasaBonga | General reference hospital | Physician | 8 |
| FG 4 | YasaBonga | Primary health centre | Head nurse | 7 |
