# Supplementary material for: Diagnostic Work-Up of Neurological Syndromes in a Rural African Setting: Knowledge, Attitudes and Practices of Health Care Providers
Source: PLoS One. 2014 Oct 23;9(10):e110167. doi: 10.1371/journal.pone.0110167 (PMC4207747; doi:10.1371/journal.pone.0110167)
Supplement: Text S1 — Check-list used for observing consultations. (DOCX) [file pone.0110167.s004.docx]

**Text S1:** Check-list used for observing consultations

case sequence number: ………………

observation date :..................................……………................................................

healthcare provider level: medical doctor □

nurse (A1) □

nurse (A2) □

consultation start time:………… consultation end time :………………

health facility: hospital:……………………… ; health centre:……………………

health zone:............................................................................................................

Province : …………...................................................................................................

Country:…………………………….....................................................................................

**1. patient reception**

1.1. Is the patient put at ease before the start of the consultation ? □yes □no

1.2. Does he question the patient directly, without attempting to build any rapport? □yes □no

1.3. Is he interested in knowing more about the patient's identify? □yes □no

1.3.1. Name: □yes □no

1.3.2. Age : □yes □no

1.3.3. Sex : □yes □no

1.3.4. Profession: □yes □no

1.3.5. Marital status: □yes □no

1.3.6. Patient residence: □yes □no

1.3.7. Other? (specify) : □yes □no

Observations :..........................................................................................................................................................................................................................................................................

**2. patient complaints**

2.1. Does he ask about the reason for the consultation? : □yes □no

2.2. Does he ask clarifications about the complaints? : □yes □no

Observations:..........................................................................................................................................................................................................................................................................

**3. patient medical history**

3.1. Ancestors

3.1.1. Grand parents ? □yes □no

3.1.2. Parents ? □yes □no

3.2. Siblings

3.2.1. Brothers? □yes □no

3.2.2. Sisters? □yes □no

3.3. Other family

3.3.1. Husband/wife? □yes □no

3.3.2. Children? □yes □no

3.4. Personal

3.4.1. Medical? □yes □no

3.4.2. Surgery? □yes □no

3.4.3. Obstetrics/Gynecology? □yes □no

3.4.4. Toxins/Allergies? □yes □no

3.4.4.1. Tobacco? □yes □no

3.4.4.2. Alcohol?  □yes □no

3.4.4.3. food or medicine allergies?  □yes □no

3.4.5. Taking medicine or traditional products ?  □yes □no

3.4.6. Religious beliefs? □yes □no

3.4.7. Eating habits? □yes □no

3.4.9. Other? (specify): □yes □no

Observations:..........................................................................................................................................................................................................................................................................

**4. History of current illness**

Does he ask about the history of the present illness? □yes □no

Observations :………………………………………………………………………………………………………………………………………………………………………………

**5. Additional anamnesis**

5.1. General condition? □yes □no

5.2. Central nervous system? □yes □no

5.3. Cardiovascular system? □yes □no

5.4. Respiratory system? □yes □no

5.5. Digestive system? □yes □no

5.6. Urogenital system? □yes □no

5.7. Locomotor system? □yes □no

5.8. Other? (specify) □yes □no

Observations :………………………………………………………………………………………………………………………………………………………………………………

**6. Physical examination**

*If relevant, specify : Inspection (I) Palpation (P) Percussion (C) Auscultation (A)*

6.1. Does he take vital signs? □yes □no

6.1.1. Blood pressure? □yes□no

6.1.2. Pulse ? □yes □no

6.1.3. Respiratory Rate ? □yes □no

6.1.4. Temperature? □yes □no

6.1.5. Weight? □yes □no

6.2. Head and neck? □yes □no specify: .....

6.3. Tegument? □yes □no specify: .....

6.4. Thyroid? □yes □no specify: .....

6.5. Lymph nodes? □yes □no specify: .....

6.6. Lungs? □yes □no specify: .....

6.7. Heart? □yes □no specify: .....

6.8. Abdomen ? □yes □no specify: .....

6.9. Urinary and genital system? □ yes □ no specify: .....

6.10. Vessels? □ yes □ no specify: .....

6.11. Limbs / Muscles / Joints? □ yes □ no specify: .....

6.12. Neurological exam:

6.12.1. Tonus ? □yes □no

6.12.2. Motricity? □yes □no

6.12.3. Reflexes? □yes □no

6.12.4. Examination of cranial nerves □yes □no

6.13. Others? □yes □nospecify: ……………………………………………

Observations :………………………………………………………………………………………………………………………………………………………………………………

**7. Differential diagnosis**

Does he establish a list of differential diagnoses? □yes □no

Observations:…………………………………………………………………………………………………………………………………………………………………………

**8. Para-clinical examinations**

8.1. Does he request a para-clinical appraisal? □yes □no

8.2. Para-clinical tests

8.2.1. Blood? □yes □no

8.2.1.1. Biochemistry? □yes □no specify: ………………..

8.2.1.2. Hematology ? □yes □no specify: ………………..

8.2.1.3. Serology? □yes □no specify: ………………..

8.2.1.4. Immunology? □yes □no specify: ………………..

8.2.1.5. Parasitology ? □yes □no specify: ………………..

8.2.1.6. Bacteriology? □yes □no specify: ………………..

8.2.2. Urine? □yes □no specify: ………………..

8.2.3. Stool? □yes □no specify: ………………..

8.2.4. Examination of cerebrospinal fluid

8.2.4.1. Cytology ? □yes □no

8.2.4.2. Biochemistry? □yes □no

8.2.4.3. Others? □yes □no specify: ............................

8.2.5. Pleural fluid? □yes □no specify: ………………..

8.2.6. Ascites? □yes □no specify: ………………..

8.2.7. Medical imaging? □yes □no specify: ………………..

8.2.8. Other examinations? □yes □no specify: ………………..

Observations :………………………………………………………………………………………………………………………………………………………………………………

**9. Treatment**

9.1. Does he explain to the patient what the health issue is? □ yes □ no

9.2. Does he start treatment before provided with lab results? □ yes □ no

Observations :………………………………………………………………………………………………………………………………………………………………………………

**10. Clinical management**

10.1. Locally? □ yes □ no

10.2. Referred? □ yes □ nospecify + reason: ………………………….

Observations :………………………………………………………………………………………………………………………………………………………………………………

**11. Use of any clinical guidelines or protocols for the management of neurological syndrome cases**

11.1. Are there any reference documents visibly available in the hospital/health centre?

□ yes □ no- if yes, specify…………………………..

11.2. If available, does the healthcare provider use any reference documents for the management of neurological syndrome cases?

□ yes □ no

Observations :………………………………………………………………………………………………………………………………………………………………………………

**12. General observations regarding consultation (Summary and additional comments)** ………………………….………………………………………………………………

………………………………………………………………………………………………

**Initials of the observing researcher:**

**Date of observation:**
