# Supplementary material for: Diagnostic Work-Up of Neurological Syndromes in a Rural African Setting: Knowledge, Attitudes and Practices of Health Care Providers
Source: PLoS One. 2014 Oct 23;9(10):e110167. doi: 10.1371/journal.pone.0110167 (PMC4207747; doi:10.1371/journal.pone.0110167)
Supplement: Text S2 — Clinical vignette used in interviews. (DOCX) [file pone.0110167.s005.docx]

**Text S2:** Clinical vignette used in interviews

A 29 year old man, married and father of three children, who has been living in the city of Mosango since birth comes to the clinic with complaints of headaches. The family members accompanying the patient indicate that his behaviour has been out of the ordinary for the past three weeks.

**Q / What is your diagnostic approach? How would you go about identifying the problem?**

**Additional details**

*Note : If the healthcare provider requests clarifications regarding the patient's complaints, make sure to ask about the reason for this question before providing the information.*

- **Headaches:** since more than a month, continuous, diffuse andvery intense
- **Unusual behaviour** : the patient talks more than usual and can be aggresive

Follow-up prompt: Ask the healthcare provider what he would do after having clarified the patient's complaints.

**Antecedents**

*Note : If the healthcare provider requests clarifications regarding the patient's antecedents, make sure to ask about the reason for this question before providing the information.*

- **Hereditary and collateral**: nothing to report
- **Personal**: no significant morbidities in the past

Follow-up: What do you do after establishing these factors?

**History of current illness**

*Note : If the healthcare provider requests clarifications regarding the history of the patient's current condition, make sure to ask about the reason for this question before providing the information.*

- Duration of the headaches: 2 months
- Mode of occurence: insidious
- Previous actions undertaken by patient: presented to other health centre previously, treated for malaria with quinine (2x500mg/7dys).
- Evolution : aggravation of headaches and occurance of aggressiveness.

Follow-up prompt: What do you do next?

**Anamnesis - other**

*Note : Always ask why the healthcare provider is requesting the information.*

- Nervous and locomotor system? : difficulties walking, trembling extremities
- Febrile? : progressive, unregular, since more than a month
- Weightloss?:yes, observed by himself and his entourage
- Digestive, cardiovascular, urinary systems?: nothing to report

Follow-up prompt: What do you do next?

**Physical examination**

*Note : Provide healthcare provider with the outcomes of the clinical examinations he suggests, but always ask why he needs that information, to evaluate what and for which reason?*

- Blood pressure : 120 / 80 mmhg ; pulse: 110 / min ; RR : 24 / min and T° : 38°C
- Head and neck: Stiff neck at the end of flexion, ganglionic area free
- Heart: Regular tachycardia, no unusual sounds
- Lungs: Normal breathing sounds
- Abdomen : slightly bloated, supple, non-sensitive, with hepatomegaly and splenomegaly
- Neurological examination: involuntary movements in rest

**Q / What are you hypotheses about this case? Why?**

*Note : Ask specifically for the motivation for each listed potential diagnosis.*

**Q / Which tests would you have done to confirm your diagnosis?**

*Note : Enquire about the reason for requesting each mentioned test before providing results.*

- globulin count: 6400 / mm3
- FL : N 29 L 65 E 6 B 0 M 0
- VS : 43 mm/ h
- Hb : 8, 2 g %
- GE : negative
- Hct : 24 %
- CATT : positive
- CSF: clear aspects, withpleocytosis mainly lymphocytic, hyperprotidorachie, hypoglycorrhachia.

**Q / Which is the most probably diagnosis? Why?**
