# Supplementary material for: Diagnostic Work-Up of Neurological Syndromes in a Rural African Setting: Knowledge, Attitudes and Practices of Health Care Providers
Source: PLoS One. 2014 Oct 23;9(10):e110167. doi: 10.1371/journal.pone.0110167 (PMC4207747; doi:10.1371/journal.pone.0110167)
Supplement: Text S3 — Interview question guide. (DOCX) [file pone.0110167.s006.docx]

**Text S3:** Interview question guide

1. **What do you think of when you hear the term 'neurological syndrome'? What do you think of the following description proposed by other people?**

- recent convulsions?
- altered state of consciousness?
- sensorimotor deficits?
- difficulties walking?
- severe headache, meningeal signs?

1. **Would you classify the case we discussed previously as being a case of the neurological syndrome? Why?**
2. **Which infectious diseases endemic in this area would you associate with the neurological syndrome?**

- Do you experience any difficulties when attempting to diagnose a patient presenting with the neurological syndrome?
- Could you give a recent example?
- Is it difficult to come to a differential diagnosis?
- Do you have access to relevant diagnostic tools? Which ones?

1. **Are there any factors you take into account when requesting para-clinical tests?**

- Are there any tests that you would routinely request for everyone? Why? Are they recommended, by example by the management of the health facility you work in?
- Do you request para-clinical tests relevant to each of your differential diagnoses? Why?

1. **Are there any factors you take into account when prescribing medical treatment?**

- Do you usually wait for laboratory test results before prescribing treatment?
- Do you have access to the necessary drugs for the treatment of cases presenting with the neurological syndrome?

1. **Are you aware of any clinical guidelines/protocols/flowcharts (whether prepared by actors from your health facility, the health zone, provincial, national or international levels) that support your clinical management of neurological syndrome cases?**

- Which ones? Describe please.
- Do you use them?Do you feel they are practical? If no, why not?
- Are these clinical reference documents available to you in your consultation room?
- Do you consult these documents in the presence of your patients? If not, why not?
- What are your thoughts regarding the referral of patients? How is that organised? Are there any issues?
