# Supplementary material for: Diagnostic Work-Up of Neurological Syndromes in a Rural African Setting: Knowledge, Attitudes and Practices of Health Care Providers
Source: PLoS One. 2014 Oct 23;9(10):e110167. doi: 10.1371/journal.pone.0110167 (PMC4207747; doi:10.1371/journal.pone.0110167)
Supplement: Text S4 — Focus group discussion question guide. (DOCX) [file pone.0110167.s007.docx]

**Text S4:** Focus group discussion question guide

1. **We are going to discuss the 'neurological syndrome':**

- What do you think of when you hear the term 'neurological syndrome'?
- What do you think of the following description proposed by other people?
  - recent convulsions?
  - altered state of consciousness?
  - sensorimotor deficits?
  - difficulties walking?
  - severe headache, meningeal signs?

1. **Which infectious diseases might cause the neurological syndrome in a patient? Why?**

- Prompt: in your setting?
- Prompt: Meningeal Tuberculosis? Cerebral malaria? Stage 2 HAT? HIV/aids? Cryptococcosis?

1. **Could you describe your diagnostic process when someone presents to your clinic with the neurological syndrome, which you suspect to be due to an infectious cause?**
2. **Are there any factors you take into account when preparing a request for para-clinical examinations?**

- Are there any examinations that you routinely request for all patients? What would these be for? Would that be under recommendation from -for example- your health facility?
- Do you request paraclinical examinations specific to each differential diagnosis you identify? If no, why not?

1. **What challengesdo you experience in diagnosing neurological syndrome cases that you expect to be due to infectious causes?**

- *Evaluate the issues that were mentioned during the interviews* (clinical examination? laboratory? the disease itself? laboratory staff?)
- How could these issues be resolved?
- How might the diagnostic process be improved?

→ Prompt: by the availability of additional or new diagnostic tools?

1. **Are there any factors you take into account when prescribing medical treatment?**

- Do you usually wait for laboratory test results before prescribing treatment?
- Do you have access to the necessary drugs for the treatment of cases presenting with the neurological syndrome?

1. **Are you aware of any clinical reference documents that support your clinical management of neurological syndrome cases?**

*Notes for interviewer:
1. Get the participants to list the documents they know of and get them to discuss their use by talking about practical examples.
2. Prepare and bring a list of the the reference documents that were mentioned during the interviews.*

- Do you use them in your daily practice? How?
- Are they practical? If not, what are the issues?
  *(note for interviewer: evaluate the issues discussed during the interviews - bring a list)*
- Are these documents available to you in your consultation rooms?
- Would you consult these documents when the patient is present? If no, why not?

1. **How do you think such clinical reference documents for the management of neurological syndrome cases could be improved?**

*Note for interviewer: Ask examples*

- Prompt: To make them more easy to use/consult? How? Other format?
- Prompt: By making them more specific to the kind of healtcare provider using them (separate versions for the nurses, doctors, health workers?)
