# Supplementary material for: Diagnostic Work-Up of Neurological Syndromes in a Rural African Setting: Knowledge, Attitudes and Practices of Health Care Providers
Source: PLoS One. 2014 Oct 23;9(10):e110167. doi: 10.1371/journal.pone.0110167 (PMC4207747; doi:10.1371/journal.pone.0110167)
Supplement: Text S5 — Coding structure. (DOCX) [file pone.0110167.s008.docx]

**Text S5:** Coding structure

1. Knowledge about the neuro syndrome
   1. Familiarity with term
   2. Knowledge about the consequences of the neuro syndrome
   3. Knowledge regarding clinical signs associated with the neuro syndrome
      1. Agitation
      2. Intense headaches
      3. Coma
      4. Convulsions
      5. Meningeal involvement
      6. Behavioural difficulties
      7. Speaking disorders
      8. Walking disturbances
      9. Fever
      10. Other clinical signs
   4. Knowledge of differential diagnoses of the neuro syndrome
      1. Bacterial meningitis
      2. Cerebral malaria
      3. Meningeal tuberculosis
      4. Human African trypanosomiasis
      5. HIV/Aids
      6. Other differential diagnoses
         1. infections
         2. substances
   5. Knowledge examination neuro syndrome
   6. Knowledge of laboratory diagnostic workup
   7. Knowledge regarding treatment for the neuro syndrome
2. Consultation process
   1. Presentation history
      1. General
      2. Additional medical history for the neuro syndrome
      3. Epidemiology of area
   2. Clinical examination procedures for the neuro syndrome
   3. Diagnostic tests
      1. Diagnostic workup requested for the neuro syndrome
      2. Laboratory tests requested for neuro syndrome
         1. Inflammatory evaluation
         2. Biochemistry
         3. Gram staining
         4. Culture
         5. Stool/urine examination
         6. Thick film
         7. Haemoglobin
         8. Medical imagery
      3. Diagnostic workup systematically requested
      4. Omitted tests
   4. Diagnosis of the neurological syndrome
      1. Posing the clinical diagnosis of the neurological syndrome
      2. Aetiologicaldiagnosis of the neurological syndrome
   5. Treatment given for the neurological syndrome
      1. symptomatic
      2. disease specific
   6. Most important phase of consultation process
3. Treatment provided prior to laboratory confirmation
   1. rationale
4. Barriers regarding the diagnosis of the neuro syndrome
   1. Lack of facilities
   2. Lack of technical ability of lab workers
   3. Lack of electricity
   4. Lack of tools
   5. Lack of reagents
   6. Lack of patient financial resources
   7. Community perceptions regarding use of diagnostics
   8. Delay of attaining test results
5. Diagnostic approach to the neuro syndrome
6. Available laboratory diagnostic tools for the neuro syndrome
7. Factors that affect laboratory diagnosis
8. Suggestions for improving the diagnosis of the neurological syndrome
9. Barriers for the treatment of the neurological syndrome
10. Clinical reference guides
    1. Availability of reference guides
    2. Unavailability of reference guides
    3. Added value of reference guides
    4. Availability of other materials for the diagnosis of the neuro syndrome
    5. Knowledge regarding available reference guides
       1. general reference guides on the clinical management of patients
       2. reference guides on the clinical management of neuro syndrome patients
       3. 'Ordinogrammes' as clinical reference guide
       4. malaria protocol as clinical reference guide
       5. meningeal tuberculosis protocol as clinical reference guide
       6. HAT protocol as clinical reference guide
       7. HIV/AIDS protocol as clinical reference guide
       8. other protocols for the clinical management of neuro syndrome
    6. Barriers regarding the use of reference guides
       1. associated with their unavailability
       2. associated with fear of losing patient confidence
       3. associated with lack of time
       4. associated with community perceptions
       5. associated with healthcare provider perceptions
    7. Suggestions for improving the use of reference guides
11. Patient referral
    1. Referral to hospital
    2. Referral to HAT program
    3. Perceptions regarding patient referral practices
       1. When to refer
       2. Importance of the referral practices
       3. healthcare provider attitudes
       4. community attitudes
    4. Barriers associated with patient referral
       1. hospital consultation cost more expensive than health centre
       2. hospital laboratory test cost
       3. hospital cost of care more expensive than health centre
       4. hospitalisation cost of care more expensive than health centre
       5. hospitalmedicine cost considered more expensive than health centre
       6. distance to hospital
       7. linked to community perceptions
       8. lack of transportation options
    5. Suggestions regarding patient referral practices
